# Out-of-Pocket Costs and Other Determinants of Access to Healthcare for Children with Febrile Illnesses: A Case-Control Study in Rural Tanzania

**DOI:** 10.1371/journal.pone.0122386

**Published:** 2015-04-10

**Authors:** Joëlle Castellani, Borislava Mihaylova, Silvia M. A. A. Evers, Aggie T. G. Paulus, Zakayo E. Mrango, Omari Kimbute, Joseph P. Shishira, Francis Mulokozi, Max Petzold, Jan Singlovic, Melba Gomes

**Affiliations:** 1 Department of Health Services Research, School for Public Health and Primary Care (CAPHRI), Maastricht University, Maastricht, The Netherlands; 2 Health Economics Research Centre, University of Oxford, Oxford, United Kingdom; 3 Kilosa Station, National Institute for Medical Research, Kilosa, Tanzania; 4 Centre for Applied Biostatistics, Occupational and Environmental Medicine, Sahlgrenska Academy, University of Gothenburg, Gothenburg, Sweden; 5 World Health Organization consultant, Libochovany, Czech Republic; 6 UNICEF/UNDP/World Bank/WHO Special Programme for Research & Training in Tropical Diseases (TDR), World Health Organization, Geneva, Switzerland; Mahidol-Oxford Tropical Medicine Research Unit, THAILAND

## Abstract

**Objectives:**

To study private costs and other determinants of access to healthcare for childhood fevers in rural Tanzania.

**Methods:**

A case-control study was conducted in Tanzania to establish factors that determine access to a health facility in acute febrile illnesses in children less than 5 years of age. Carers of eligible children were interviewed in the community; cases were represented by patients who went to a facility and controls by those who did not. A Household Wealth Index was estimated using principal components analysis. A multivariable logistic regression analysis was performed to understand the factors which influenced attendance of healthcare facility including severity of the illness and household wealth/socio-demographic indicators. To complement the data on costs from community interviews, a hospital-based study obtained details of private expenditures for hospitalised children under the age of 5.

**Results:**

Severe febrile illness is strongly associated with health facility attendance (OR: 35.76, 95%CI: 3.68-347.43, p = 0.002 compared with less severe febrile illness). Overall, the private costs of an illness for patients who went to a hospital were six times larger than private costs of controls ($5.68 vs. $0.90, p<0.0001). Household wealth was not significantly correlated with total costs incurred. The separate hospital based cost study indicated that private costs were three times greater for admissions at the mission versus public hospital: $13.68 mission vs. $4.47 public hospital (difference $ 9.21 (95% CI: 7.89 -10.52), p<0.0001). In both locations, approximately 50% of the cost was determined by the duration of admission, with each day in hospital increasing private costs by about 12% (95% CI: 5% - 21%).

**Conclusion:**

The more severely ill a child, the higher the probability of attending hospital. We did not find association between household wealth and attending a health facility; nor was there an association between household wealth and private cost.

## Introduction

While malaria as a cause of severe childhood febrile illnesses declines, recent research emphasises the prevalence of other causes of infection as primary causes of fever and the importance of these infections in hospital admissions, especially in Africa [1–4]. Fevers appear to have been consistently over-diagnosed and treated as malaria [2, 3] sometimes with adverse outcomes [5].

In most severe febrile illness, speed of therapeutic intervention determines outcome. By definition, severely ill children have declared themselves as being unable to control the infection and have a lower probability for spontaneous cure. While uncomplicated illness may be managed at home, the evolution of an infection to a severe state or death suggests failure to diagnose and promptly treat the infection. Whereas a negative relationship has been reported between poverty and use of health services for uncomplicated febrile illness [6], the relationship between wealth and healthcare seeking behaviour in severe disease remains almost unexplored, except for one study which showed no association [7].

In rural areas, it has been reported that irrespective of illness, 41% Tanzanian patients consult a healthcare provider when sick, and among those with “danger” signs of fast or difficult breathing, convulsions, inability to eat, drink or suck, repeated vomiting, altered consciousness or coma, 60% go to a public hospital/health centre [6]. In order to decrease private costs and encourage greater and more equitable use of health services among children under 5 years old who bear the brunt of mortality, this group are exempt from healthcare charges, including inpatient fees [8]. Despite this policy, approximately 75% of health care expenditure appears to be borne by households [9] and high costs for hospital paediatric admissions are reported [10].

Reasons why households fail to reach a health facility are still not very clear. Anticipated total private costs, inability to pay in kind or pay over a longer period of time may be some of the reasons [11].

Understanding failed opportunities to access timely care is key to reducing the infectious disease burden. Some part of the failed opportunity may be due to delays in taking a decision to reach care. Another part may be the delay or failure of the family to reach appropriate care. The third delay may occur at the facility when clinicians do not have a high index of suspicion for the true cause in their diagnostic and treatment plan.

This study looks at determinants of access to health care for children with febrile illness. We carried out a case-control study in rural Tanzania, where malaria, acute respiratory infections and other causes of serious febrile illness are common, in order to understand whether economic and socio-demographic factors determine access to healthcare facilities.

## Methods

### Study sites

Our study was implemented in two rural, malaria-endemic areas of Tanzania: Kilosa and Mvomero Districts, between September 2010 and March 2011. In Kilosa District, there is access to free care at a public hospital, and most families farm on their own land. In contrast, family income in Mvomero District is dependent upon large-scale, estate-owned, irrigated sugarcane and rice to which families contribute labour. The extensive irrigation makes malaria transmission intense and non-seasonal compared with surrounding areas [12]. Access to immediate care is through a mission hospital located close to the irrigated areas that charges fees.

### Health services provision in study areas

At Kilosa District Hospital, facility registration/consultation, bed costs, inpatient cost of drugs and laboratory examinations are provided free of charge for young children. However, for older children and adults, a fixed fee of US$1.4 is charged for registration/consultation/ medication plus $0.3 per night per bed and per test for each laboratory investigation. In public hospitals such as Kilosa District Hospital, drugs or other supplies used for patient care are provided at no cost when they are in stock. However if essential commodities happen to be out-of-stock at the time of the consultation/admission, the responsibility for purchase rests with the patient’s carer.

Turiani Missionary Hospital in Mvomero District was set up in 1961 to provide medical services labourers of the local irrigation schemes [13]. Young children are charged $2.4 for the first visit for registration/consultation plus $0.7 per bed per night, $1.2 for the second visit plus $0.7 per night per bed. The cost for older children and adults is double that of Kilosa: $2.8 for registration/consultation plus $0.7 per night per bed. The cost of drugs and laboratory examinations are charged separately, per test and per drug.

Although costs charged to the patient are different between public and private facilities, both hospitals follow national treatment guidelines and provide the same standard of care. The number and quality of staff attending patients were not noticeably different between the two hospitals, but greater attention seemed to be paid to patient notes, hospital files, documentation of patient care and follow up in Turiani Missionary Hospital.

These hospitals serving both Districts represent the highest level of care; they are supported by several other public and private facilities (called health centres and dispensaries) which provide inpatient and outpatient health care respectively. The dispensary managed by nurses is usually the first point of consultation for patients and they are closer to the patients’ residences and are more numerous than hospitals. The health centre admits patients, and is staffed by doctors and nurses and often located at an intermediate distance between the dispensary and hospital. In addition, there are a variety of private laboratories, governmental maternal-care clinics, traditional healers and voluntary health workers to whom patients can go for advice and care, in addition to the shops which sell medicines (antipyretics, antibiotics, and antimalarials including quinine) without prescription.

### Study population and data collection

The case-control study was carried out in Kilosa and Mvomero Districts. The purpose was to establish why some parents or carers went to hospital/health centre with their child and others did not, especially for children who had a severe febrile illness. A list was developed identifying the villages where the majority of hospital/health-centre-admitted patients resided (excluding villages next to those facilities) and community interviews occurred in the twelve top communities listed, so that geographical distance was similar for patients admitted or not admitted to these facilities.

In each community, the study was explained to chiefs of villages and community health workers (CHWs) who are the front-line workers for community health care. These CHWs are required to keep a ledger of sick children in the community with their symptoms, consultations and outcome. We outlined our desire to interview parents of sick children focusing on those who had symptoms of a febrile illness in the past month. CHWs were approached to identify patients who met the inclusion criteria of age (between 3–59 months), symptoms (a febrile illness which prevented oral drug intake at some point during the illness), and illness resolution within the past month.

Children who did not meet these eligibility criteria, whose guardian was not present during the illness, who had been already interviewed regarding a previous episode of illness in the household or who refused to sign the informed consent form were excluded from interview. Once eligible children were identified by village health workers or village chiefs, the parents or guardians of the children were approached, the study was explained, informed consent obtained, and interviews were conducted.

Allocation of the participants to the cases or controls and determination of whether the episode met eligibility criteria could only occur post-interview. Cases were defined as children who had attended a health facility (i.e. hospital or health centre) and controls were those who did not.

Thirteen trained interviewers, with extensive research experience in Kilosa, carried out structured interviews in Swahili and filled out a Case Record Form (available on request). There was no purposeful selection of families for interviews: in each village, all families identified with a sick child who met the inclusion criteria were interviewed. Only one interview per household occurred even if many children in the household had been sick, or a child had more than one episode of illness during the study period.

To better understand the factors driving hospital costs, a complementary study was carried out in hospitals with a focus on all out-of-pocket costs of patients admitted, as well as the clinical history of children prior to admission. These interviews were carried out with parents of children about to be discharged from Kilosa District Hospital and Turiani Mission Hospital. Interviews occurred on the day of discharge so that most costs already incurred by parents could be captured. When many children were discharged on the same day, a selection of parents for interview was undertaken (first discharged, first interviewed); when only a few eligible children were to be discharged, every guardian (i.e. an adult carer accompanying the child) was interviewed.

### Sampling strategy

Our aim was to interview all patients meeting the eligibility criteria from the main 12 malaria endemic communities in the catchment areas of two major hospitals and one public health centre using CHWs records of childhood illnesses in the community. All families of a child meeting the eligibility criteria were interviewed. At intervals, after a visit to a village had already taken place, the CHW would inform the team that new patients had been identified, and the team would return to the village to complete additional interviews. The objective was to fully represent all eligible patients in these communities during the malaria season of September 2010 to March 2011. There was no attempt to have an equal number of patients from each of the 12 communities.

### Questionnaire design

All questionnaires were in Swahili and pilot tested before use. The hospital questionnaire was similar to the community questionnaire but captured additional information on the date and time of arrival of the patient at the hospital, the clinical diagnoses and the treatments received/prescribed at the hospital, extracted from the patient’s hospital file. Each interview lasted about fifty minutes. Participants were asked about the general social-demographic context of the family, and detailed information of the clinical course of the illness (timing, symptoms, actions taken, healthcare providers visited and costs incurred such as transportation, medicines, registration/consultation fees, laboratory/diagnostic tests, accommodation and food for each consultation).

### Data analysis

#### Patient and household characteristics

Demographic information on the patient (sex, age) and patient’s family (education, number of working members) was obtained. Household socioeconomic data focussed on living standards—durable family possessions (radio, lantern, bicycle, table, iron), ability to meet family food needs, and main occupation/means of the household. Baseline characteristics were compared between cases and controls.

#### Severity of illness

Classification of severity was by clinical symptoms as reported by the caretaker. Febrile children with reports of only some very short period of time when the child could not take oral drugs and where the child was largely able to take oral medications, were classified as *Per Os* (PO). This category included children who had fever only, diarrhoea, rash, cough, a cold/runny nose, headache, no appetite or abdominal pain. If the fever was accompanied by one of the following: repeated vomiting or lethargy (unable to sit/stand/walk unaided, too weak to eat, drink or suck) the illness was classified as *Non-Per-Os* (NPO). Children with repeated convulsions, altered consciousness or coma, difficulties in breathing or rapid breathing, a stiff neck, bulging fontanel or chest indrawing were classified as severely ill. There was no overlap in patients; children with symptoms in more than one category were categorised in the highest severity category.

#### Private out-of-pocket costs

Out-of-pocket costs reported by the parent or guardian of the child were categorised into “hotel” costs (defined as accommodation costs, registration costs, food, drinks and other costs for carer or patient), diagnostic/laboratory investigations, drugs and patient management, and transport. Transport cost included costs of the parent or guardian but excluded costs paid by a third party (i.e. a person not related to the household) accompanying the parent/guardian and child. Total private costs were compared by case-control status for each location. Total hospital costs were compared by location also for the patients interviewed at the hospital. Costs are presented in US dollars ($) using the average exchange rate between September 2010 and March 2011: 1 US Dollar = 1,474.06 Tanzanian Shillings (www.oanda.com).

#### Statistical methods

All data were double entered (Epidata, 3.1) and analyzed using STATA v.9.2 (StataCorp, College Station, TX, USA). Information on household possessions (table, radio, lantern, bicycle and iron) and food problems (had or never had food problems) was used to calculate a Household Wealth Index (HWI) based upon principal components analysis to characterize the wealth variance between households within the community group [14, 15]. Households were grouped into pre-determined ‘wealth’ categories—the lowest 40%, middle 40% and highest 20%—reflecting different socioeconomic levels. Calculation of the HWI did not adjust for household size since the benefits of possessions would be available at the household level.

Since one of our objectives was to study factors influencing hospital care, we undertook a multivariable logistic regression analysis using community-based data in which attendance at a hospital or health centre was the dependent variable and independent variables were demographic, social or economic in nature—location, age of the child, gender, highest education (in years) achieved within the family, number of working people in the household, severity of the illness and the household wealth index. Since the total private costs of illness were not known at the time of decision to go to a hospital/health centre, we excluded total private costs incurred for healthcare of the child during the episode of illness. A further linear regression analysis was used to determine which factors affected hospital costs.

When the study population was stratified with respect to the characteristic of interest, we used either the chi-squared test of homogeneity or a linear trend of odds if there were more than two ordered groups. We also used the student’s t-test to determine equality of means. The level of significance of p = 0.05 and a confidence level of 95% were used throughout.

### Ethics

The research protocol was approved by the National Institute for Medical Research Ethics Committee (NIMR) and the Commission for Science and Technology (COSTECH) in Tanzania. Additional local permission was granted by the Regional Medical Officer in Morogoro, the District Medical Officers in Kilosa and Mvomero and village leaders. Individual written informed consent was obtained from all participants prior to interview.

## Results

### Patient characteristics

**Fig 1:**
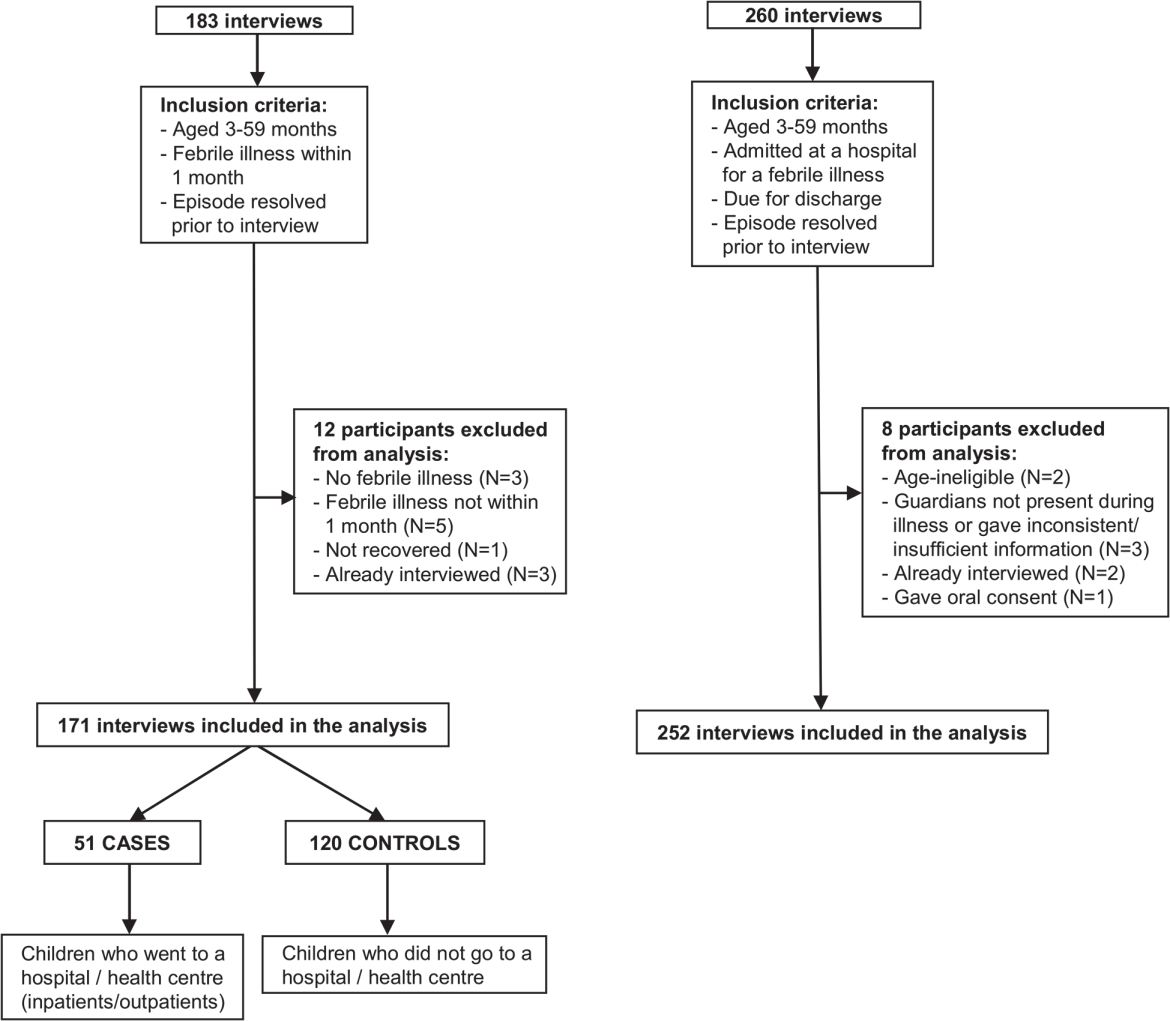
Community interviews—Cases and Controls (A); Hospital interviews (B).

Fig 1 shows the number of interviews conducted both in the community (Fig 1A) and at the hospital (Fig 1B), and the inclusion and exclusion of the data into the analysis. For our case-control study (Fig 1A), a total of 183 guardians, mostly mothers (>90%), were interviewed in the community, but 12 interviews were excluded from analysis because the child was sick at the time of the interview (N = 1), did not have a febrile illness (N = 3) or the illness had not resolved within the month’s eligibility period (N = 5). Also excluded were 3 episodes in children whose guardians were interviewed twice for different illness episodes; only the first interview was retained (N = 3). This left a total of 171 participants included in the case-control analysis (51 cases and 120 controls).

Fig 1B shows that out of 260 hospital interviews conducted to obtain detailed private costs for childhood admissions, 252 observations were included in the analysis. Eight patients were excluded: because the child was age-ineligible (N = 2); guardians were interviewed for 2 different illness episodes from which only the first was retained (N = 2); the guardian gave insufficient or inconsistent information (N = 3) or guardian gave oral but not written consent (N = 1).

### Baseline characteristics

Most children were below 37 months of age (84.4% Cases Kilosa, 73.7% Cases Turiani, 71.2% Controls Kilosa and 75.0% Controls Turiani) (Table 1). The highest level of education completed by a household member was generally primary school (7 years or less) and for most households income was based on farming (mainly crops but sometimes pastoral farming). There was no difference in baseline characteristics between cases and controls in Kilosa. In Turiani, a significantly greater proportion of working people were in households of the cases compared with controls (16/19 vs. 8/16; p = 0.0323). None of the controls were referred to a hospital and only 5 cases (2.9%) were referred to a hospital/health centre.

**Table 1:** Baseline characteristics: cases versus controls by location.

Category	Subcategory	Cases				Controls					
		Kilosa		Turiani		Kilosa		Turiani		TOTAL	
		%	N	%	N	%	N	%	N	%	N
		18.7	32	11.1	19	60.8	104	9.4	16	100.0	171
**Caregiver’s gender**	Female	93.8	30	100.0	19	93.3	97	93.8	15	94.2	161
**Child’s gender**	Male	43.8	14	52.6	10	51.9	54	37.5	6	49.1	84
Child’s age*	≤18 months	40.6	13	52.6	10	42.4	44	37.5	6	42.7	73
	19–36 months	43.8	14	21.1	4	28.8	30	37.5	6	31.6	54
	37–59 months	12.5	4	26.3	5	28.8	30	25.0	4	25.1	43
Highest education in family (years)**	No education	-	-	5.3	1	4.8	5	-	-	3.5	6
	≤ 7 years	93.8	30	68.4	13	82.7	86	87.5	14	83.6	143
	> 7 years	6.2	2	26.3	5	12.5	13	6.3	1	12.3	21
**Number of working people §**	≤ 2	50.0	16	15.8	3	61.5	64	50.0	8	53.2	91
	> 2	50.0	16	84.2	16	38.5	40	50.0	8	46.8	80
**Main occupation/source of income**	Only farming	96.9	31	68.4	13	92.3	96	81.2	13	89.5	153
	Self-employment mainly	-	-	10.5	2	5.8	6	12.5	2	5.8	10
	Paid employment mainly	3.1	1	21.1	4	1.9	2	6.3	1	4.7	8
**Symptoms & severity**	***Per Os* (PO)—Total**	**59.4**	**19**	**10.5**	**2**	**55.8**	**58**	**56.2**	**9**	**51.5**	**88**
	Fever only	21.0	4	50.0	1	50.0	29	44.5	4	43.2	38
	Diarrhoea	15.8	3	50.0	1	10.3	6	22.2	2	13.6	12
	Cough/cold	63.2	12	-	-	34.5	20	33.3	3	39.8	35
	Rash	-	-	-	-	5.2	3	-	-	3.4	3
	***Non Per Os* (NPO)—Total**	**25.0**	**8**	**84.2**	**16**	**43.2**	**45**	**43.8**	**7**	**44.4**	**76**
	Repeated vomiting +	75.0	6	87.5	14	77.8	35ǂ	71.4	5	78.9	60
	Lethargy (too weak to swallow, to sit) +	25.0	2	12.5	2	22.2	10	28.6	2	21.1	16
	**Severe—Total**	**15.6**	**5**	**5.3**	**1**	**1.00**	**1**	**-**	**-**	**4.1**	**7**
	Altered consciousness / coma	-	-	-	-	100.0	1	-	-	14.2	1
	Convulsions	40.0	2	100.0	1	-	-	-	-	42.9	3
	Difficulties in breathing	60.0	3	-	-	-	-	-	-	42.9	3
**Cases referred by a village health worker or a dispensary to hospital/health centre**	PO	-	-	-	-	-	-	-	-	-	-
	NPO	6.2	2	15.8	3	-	-	-	-	2.9	5
	Severe	-	-	-	-	-	-	-	-	-	-

* Cases Kilosa: data on 1 participant is missing.

** Controls Turiani: data on 1 participant is missing.

§ Cases Turiani vs. Controls Turiani (test of homogeneity): χ^2^ = 4.58, p = 0.0323.

+ Plus other symptoms preventing oral treatment.

ǂ Repeated vomiting only: 1 participant.

### Household Wealth Index

Table 2 presents the Household Wealth Index (HWI). Households in the lowest wealth category were least likely to have all the possessions and had a greater frequency of food problems and *vice versa*. Wealth comparisons between cases and controls using the derived HWI show that there was a non-significant trend towards more cases than controls being in the higher wealth category in both Kilosa (25.0% Cases vs. 18.3% Controls) and Turiani (21.1% Cases vs. 12.5% Controls) (Table 2).

**Table 2:** Household Wealth Index for the community participants.

	Wealth category derived from Household Wealth Index*					
Study participants (%) by wealth category	Lowest (Lowest 40%) (n = 69)		Middle (Middle 40%) (n = 69)		Highest (Upper 20%) (n = 33)	
	%	N	%	N	%	N
**Cases Kilosa**	43.7	14	31.3	10	25.0	8
**Cases Turiani**	42.1	8	36.8	7	21.1	4
**Total Cases**	43.2	22	33.3	17	23.5	12
**Controls Kilosa**	39.4	41	42.3	44	18.3	19
**Controls Turiani**	37.5	6	50.0	8	12.5	2
**Total Controls**	39.2	47	43.3	52	17.5	21

*Estimated using information on household possessions (table, radio, lantern, bicycle and iron) and food problems.

### Determinants of attending hospital or a health centre for febrile illness

Using a multivariable logistic regression (Table 3) we studied factors that might determine healthcare facility attendance. The results of this regression model indicate that only 3 independent variables significantly influenced facility attendance: location, the number of working people in the household and severity of illness. Children in Turiani (OR: 3.55, 95% CI: 1.48–8.51, p = 0.005) and children living in households with more than two working people (OR: 2.25, 95% CI: 1.03–4.95, p = 0.043) were more likely to attend a health facility than children in Kilosa or children from households with fewer working people. Using our classification of severity, the more severe the episode, the greater was the likelihood that the child would be taken to a health facility (OR: 35.76, 95% CI: 3.68–347.43, p = 0.002), although the confidence interval is very wide. The index of household wealth was neither significant as an independent covariate, nor positively correlated with total costs (S1 Table).

**Table 3:** Determinants of attending healthcare facility for acute febrile illness based on a multivariable logistic regression model (169 participants).

Variable *	Covariates	Odds ratio	95% CI	p-value
Community	Kilosa	reference		
	Turiani	3.55	1.48–8.51	0.005
Age of child (months)	-	0.98	0.95–1.00	0.080
Gender	Male	reference		
	Female	1.21	0.57–2.58	0.617
Highest education in family (years)	-	1.08	0.90–1.29	0.405
Number of working people	≤ 2	reference		
	> 2	2.25	1.03–4.95	0.043
Severity of febrile episode	*Per Os*	reference		
	*Non Per Os*	1.55	0.69–3.48	0.287
	Severe	35.76	3.68–347.43	0.002
Wealth index **	-	0.99	0.76–1.28	0.942

* Total private costs of illness were excluded from the regression analysis since these were not known at the time of parental decision to go to a hospital/health centre. Its inclusion did not affect observed relationships.

** The higher the score, the better the wealth.

### Household private out-of-pocket costs for healthcare

Table 4 presents reported private costs for a whole episode of illness by cost category, for cases and controls, by location.

**Table 4:** Mean private costs (US Dollar) for a whole episode of an acute febrile illness by case-control status and location.

	N	“Hotel” cost (SD)*	Diagnosis & laboratory cost (SD)	Drug & treatment cost (SD)	Transport cost (SD) †	(N)	No. admissions, Duration of admission in days (SD)	TOTAL Private Cost (SD)	Comparison of total private cost Cases vs Controls: p-value
**Cases—Kilosa**	32	0.19 (0.46)	0.10 (0.57)	0.45 (0.87)	0.64 (1.77)	32	5 admissions, 1.91 (1.19)	1.37 (2.26)	
**Cases—Turiani**	3ǂ	5.28 (4.93)	1.02 (0.34)	3.35 (1.56)	1.36 (1.36)	19	15 admissions, 3.17 (0.79)	12.93 (5.94)	
**Total Cases**	35ǂ	0.63 (1.93)	0.18 (0.61)	0.70 (1.23)	0.70 (1.73)	51	20 admissions, 2.85 (1.04)	5.68 (6.91)	
**Controls—Kilosa**	104	0.00 (0.03)	0.13 (0.55)	0.50 (0.91)	0.16 (0.37)	104	-	0.79 (1.44)	Kilosa: p = 0.0874
**Controls—Turiani**	15ǂ	0.25 (0.56)	0.01 (0.05)	0.92 (1.01)	0.32 (0.85)	16	-	1.62 (1.39)	Turiani: p<0.0001
**Total Controls**	119ǂ	0.03 (0.21)	0.11 (0.52)	0.55 (0.93)	0.18 (0.45)	120	-	0.90 (1.46)	All Cases vs. Controls: p<0.0001

* Includes registration and consultation costs, bed costs, food costs and other costs such as soap, toilet paper, etc.

† Transport cost included only for the guardian.

ǂ Excludes 17 participants (16 Cases and 1 Control in Turiani) unable to provide detailed costs by cost category; their costs are included in Total costs.

Cases Turiani vs. Cases Kilosa: p<0.0001 for difference in private cost.

Controls Turiani vs. Controls Kilosa: p = 0.0352 for difference in private cost.

Patients who attended a hospital/health centre always had higher private costs: $1.37 vs $0.79 (difference $0.58 (95% CI: -0.09–1.25), p = 0.0874) for Kilosa; $12.93 vs. $1.62 for Turiani (difference $11.31 (95% CI: 8.22–14.41), p<0.0001) with the difference in cost between cases and controls significant only in Turiani. Hence the private costs of patients who went to a hospital were six times larger than private costs of controls ($5.68 vs. $0.90, p<0.0001). Patients who went to Turiani Missionary Hospital spent about 9.4 times more than patients who attended Kilosa facilities $12.93 vs. $1.37, (difference $11.56 (95% CI: 9.22–13.90), p<0.0001) and had a longer duration of hospital admission (3.17 days for Turiani vs. 1.91 days for Kilosa). A separate analysis of private costs for each wealth category for cases in Kilosa and Turiani (S1 Table) confirm significant cost differences by location, and found a significant trend in the total private costs paid by wealth category only in Kilosa (p = 0.042).

Table 5 uses data from the hospital interviews to classify the symptoms reported by the caretaker for children admitted and shows similar proportions of children with mild infections admitted, but a greater proportion of admissions for severe illnesses in Turiani than in Kilosa 28.9% vs 19.1%. Table 6 explores in detail the components of hospital costs stratified by severity of illness for these patients. In both hospitals, the “hotel” costs of admission drove private costs, being approximately 50% of total costs incurred. Furthermore, the drugs and treatment costs in Turiani hospital represent more than 30% of the total hospital costs ($4.50/13.68) while in Kilosa hospital, these constituted a more modest 9% ($0.41/4.47). Transport costs were similar in both locations but constituted a relatively higher proportion of private costs for Kilosa admissions. Finally, for those who were admitted, there was no trend of increasing duration of admission or total private hospital costs with illness severity. In a generalized linear model assessing the independent factors contributing to hospital costs for admitted patients (S2 Table), the number of days of admission was a significant determinant of private cost, with each day in hospital increasing private costs by about 12% (95% CI: 5%- 21%). The data confirm that patients admitted at Turiani hospital spent more than patients at Kilosa hospital: $13.68 Turiani vs. $4.47 Kilosa (difference $9.21 (95% CI: 7.89–10.52), p<0.0001) (Table 6) with the private costs of those admitted in Turiani about 3 times higher than those admitted in Kilosa (multiplicative effect 3.1 (95% CI: 2.5–3.9; S2 Table).

**Table 5:** Symptoms and severity of acute febrile illness for the participants interviewed at the hospital.

Category	Subcategory	Hospital			
		Kilosa (N = 162)		Turiani (N = 90)	
		%	N	%	N
Symptoms & severity	***Per Os* (PO)—Total**	**19.8**	**32**	**20.0**	**18**
	Fever only	37.5	12	27.8	5
	Diarrhoea	28.1	9	33.3	6
	Cough	28.1	9	33.3	6
	Other febrile (headache, no appetite, abdominal pain)	6.3	2	5.6	1
	***Non Per Os* (NPO)—Total**	**61.1**	**99**	**51.1**	**46**
	Repeated vomiting +	86.9	86ǂ	89.1	41
	Lethargy (too weak to swallow, to sit) +	13.1	13	10.9	5
	**Severe—Total**	**19.1**	**31**	**28.9**	**26**
	Altered consciousness / coma	-	-	15.4	4
	Convulsions	61.3	19	23.1	6
	Difficulties in breathing	32.3	10	57.7	15
	Other danger signs (Stiff neck, bulging fontanel, chest indrawing)	6.4	2	3.8	1

+ Plus other symptoms preventing oral treatment.

ǂ Repeated vomiting only: 1 participant.

**Table 6:** Mean private hospital costs (US Dollar) and duration of admission by location and illness severity for participants interviewed at hospital.

	N	Duration of admission. days (SD)	“Hotel” cost (SD)*	Diagnosis & laboratory cost (SD)	Drug & treatment cost (SD)	Transport cost (SD) †	TOTAL PRIVATE COST (SD)	Comparison of total private cost, Turiani vs. Kilosa, p-value
Kilosa	162	3.02 (1.62)	2.42 (2.29)	0.01 (0.06)	0.41 (1.68)	1.64 (3.01)	4.47 (4.57)	
*Per Os*	32	2.71 (1.18)	2.81 (3.00)	0.00 (0.00)	0.21 (0.55)	1.43 (2.29)	4.45 (4.77)	
*Non Per Os*	99	3.15 (1.81)	2.39 (2.03)	0.00 (0.03)	0.52 (2.07)	1.61 (2.69)	4.52 (4.45)	
Severe	31	2.93 (1.31)	2.14 (2.24)	0.02 (0.12)	0.25 (0.81)	1.93 (4.41)	4.34 (4.91)	
Turiani	90	2.76 (1.49) ≠	6.65 (3.37)	1.02 (0.64)	4.50 (2.38)	1.51 (2.21)	13.68 (5.87)	p<0.0001
*Per Os*	18	2.82 (1.61)	6.81 (3.69)	1.17 (0.65)	4.29 (2.19)	1.26 (1.94)	13.52 (6.63)	p<0.0001
*Non Per Os*	46	2.71 (1.08) ≠	6.63 (2.75)	0.97 (0.65)	4.56 (2.19)	1.37 (1.63)	13.53 (4.66)	p<0.0001
Severe	26	2.80 (1.99)	6.57 (4.20)	1.02 (0.64)	4.54 (2.87)	1.92 (3.14)	14.05 (7.31)	p<0.0001

* Includes registration and consultation costs, bed costs, food costs and other costs such as soap, toilet paper, etc.

† Transport cost included only for the guardian.

≠ Missing for 1 participant

Trend p-value for total private costs by episode severity in Kilosa: p = 0.93.

Trend p-value for total private costs by episode severity in Turiani: p = 0.79.

## Discussion and Conclusion

In this study of determinants of access to health care for acutely ill children, severity of symptoms was the most important predictor of attending a hospital facility. Household wealth did not appear to influence this decision, although, as expected, total costs increased with admission and the number of days admitted at a facility.

These findings contrast with reports of surveys implemented almost 20 years ago, when 40% of young children with an acute febrile illness were reported to have died without contact with health services [16], and intermediate studies which indicated that 40% of children with danger signs were not taken to appropriate care and this tended to be worse in poorer families [6]. Thus our finding that symptoms, not wealth, was decisive in the decision to proceed to a hospital is both reassuring and indicative of behavioural changes in the management of serious paediatric infections that may have taken place more recently [7].

Most of our participants—cases (72.5%) and controls (59.2%)—indicated that they had incurred some out-of-pocket expenses and for those who went to hospital, our complementary study indicated that total costs incurred were linked to hospital type with approximately half of private costs for hospitalised patients being related to the food and accommodation costs of the child and guardian. The average expenditure at the public hospital was comparable with the average of $5.5 reported in a review of private costs of paediatric hospital admissions carried out in Tanzania the year before our study [10]. However, unlike the review’s findings on church-based hospitals, we found substantially greater private costs incurred at the mission hospital we studied. We note that Turiani Mission Hospital had not yet acquired a “designated district hospital” status, which would normally enable such a hospital to be jointly subsidized by the Government as well as private church-based sources. Designated status also requires the hospital to follow the same policy of free provision of care for children (including drugs, laboratory expenses and food) as public hospitals, except for a patient-file fee at the time of admission. Hence the absence of the designated status meant that a substantial proportion of the costs of mission hospitalization were passed onto patients. The three-fold higher out-of-pocket average costs for the mission hospital lasted until 2012 when the hospital acquired the designated district hospital status.

Although mean hospitalization payments were lower in the government hospital compared with the mission hospital, poorer patients attending either hospital did not spend more on the episode compared with their wealthier counterparts. Costs for a child who had a severe febrile illness and was admitted at the public hospital were approximately 16.5% of the average monthly income of $27.14 [17] while admission at the mission hospital meant a loss of about 50% of average monthly income. Assuming an incidence of 3.5 episodes of malaria for a child under 5 years old per year [18], health costs would have been about 5% of family income per child in the Kilosa area and about 15% in the Turiani area. This supports the finding of Saksena et al. [10] which suggests that around 71% of households lose income due to a child’s admission at a health care facility. We did not obtain data on how families paid for their out-of-pocket costs, but reviews have shown that the immediate response is to use available cash and mobilise savings (which is only feasible for a small proportion of households) whereas other households reduce consumption (often food) or sometimes sell assets—that form an important source of the family’s livelihood, such as land or livestock, or borrow from social networks, which can place them in debt for some period of time [19].

Our study has several limitations. First, we had assumed that the cases in Turiani and Kilosa could be pooled for the cost analysis; we did not anticipate the degree of cost heterogeneity that we found which required sub-group analyses. Secondly, because of the prior literature we anticipated a far larger number of patients with severe episodes who did not proceed to the hospital and thus a larger number of controls. In the event, from our patient sample with acute symptoms, less than 10% had severe symptoms (altered consciousness or convulsions) and all of them except for one participant went to hospital. Thirdly, we used a pragmatic symptomatic classification of patients into different categories of disease severity. This classification met WHO integrated management of childhood illness (IMCI) guidelines [20, 21]. Children with danger signs (convulsions, repeated vomiting, failure to feed, lethargy/unconsciousness, chest in-drawing, noisy breathing, severe dehydration, and pallor) are referred to nearby health facilities. For our analysis, we classified the children with danger signs into two groups. Those who had repeated convulsions, altered consciousness or coma, difficulties in breathing or rapid breathing, a stiff neck, bulging fontanel or chest in-drawing were classified as severely ill, those who had repeated vomiting or lethargy (unable to sit/stand/walk unaided, too weak to eat, drink or suck) were classified as *Non-Per-Os*. There is an obvious overlap between these two groups and misclassification between these categories might have occurred.

There is a high probability that we have underestimated total private costs. Return journeys to the hospital were not taken into account in the costs of participants interviewed at the hospital and although other studies have doubled the transport costs, we judged that transport costs to the hospital in an emergency would be higher than the costs of the return journey being undertaken in more favourable circumstances. Once the child had recovered and was on the point of discharge, participants indicated that they were not sure about the mode of transport they would use to go back home, or the costs associated with this journey but did not anticipate the costs to be as high as the journey to hospital. In addition, drugs used in home management of the episode, and perhaps bought during a previous occurrence of illness were not included in our tally of total costs because neither the amount of drug used nor the cost could be determined. We also excluded the opportunity costs of time since our study concentrated on direct out-of-pocket costs. Finally the cost data from the cases or controls would have been based upon recall of expenditures made since the episode and there are likely to be errors in recall of expenditures made, although we anticipate that the magnitude of the errors might be similar between the cases and controls.

Our focus was on whether economic and socio-demographic variables played a role in accessing a hospital or health centre. Therefore, other factors that would influence behaviour, such as perception of illness and parental understanding of health needs in an emergency, qualitative data on perceptions about quality of care and services, staff attitude and competencies at the health facility were not sought. Such qualitative data might have been informative on choice and timing of care. Finally, although we would have wanted to investigate characteristics of missed opportunities—referred participants who did not comply with referral advice—none of our controls said that they had been referred to the hospital. Thus, supplementary work on failed referral may complement our findings.

## Supporting Information

S1 Table Mean private costs (US Dollar) for a whole episode of febrile illness for the cases by location, stratified by household wealth category. (DOC)

S2 Table Determinants of private hospital costs for an episode of febrile illness based on a generalized linear model for 249 participants interviewed at the hospital. (DOC)

## References

[1] Crump JA, Morrissey AB, Nicholson WL, Massung RF, Stoddard RA, Galloway RL, et al Etiology of severe non-malaria febrile illness in Northern Tanzania: a prospective cohort study. PLoS Negl Trop Dis. 2013; 7: e2324 10.1371/journal.pntd.0002324 23875053 PMC3715424

[2] D'Acremont V, Kilowoko M, Kyungu E, Philipina S, Sangu W, Kahama-Maro J, et al Beyond malaria—causes of fever in outpatient Tanzanian children. N Engl J Med. 2014; 370: 809–817. 10.1056/NEJMoa1214482 24571753

[3] Maltha J, Guiraud I, Kabore B, Lompo P, Ley B, Bottieau E, et al Frequency of severe malaria and invasive bacterial infections among children admitted to a rural hospital in Burkina Faso. PLoS One. 2014; 9: e89103 10.1371/journal.pone.0089103 24551225 PMC3925230

[4] Church J, Maitland K. Invasive bacterial co-infection in African children with Plasmodium falciparum malaria: a systematic review. BMC Med. 2014; 12: 31 10.1186/1741-7015-12-31 24548672 PMC3928319

[5] Reyburn H, Mbatia R, Drakeley C, Carneiro I, Mwakasungula E, Mwerinde O, et al Overdiagnosis of malaria in patients with severe febrile illness in Tanzania: a prospective study. BMJ. 2004; 329: 1212 15542534 10.1136/bmj.38251.658229.55 PMC529364

[6] Armstrong Schellenberg J, Victora CG, Mushi A, de Savigny D, Schellenberg D, Mshinda H, et al Inequities among the very poor: health care for children in rural southern Tanzania. Lancet. 2003; 361: 561–566. 12598141 10.1016/S0140-6736(03)12515-9

[7] de Savigny D, Mayombana C, Mwageni E, Masanja H, Minhaj A, Mkilindi Y, et al Care-seeking patterns for fatal malaria in Tanzania. Malar J. 2004; 3: 27 15282029 10.1186/1475-2875-3-27 PMC514497

[8] Mubyazi GM. The Tanzanian Policy on Health-care fee waivers and exemptions in practice as compared with other developing countries: Evidence from recent local studies and international literature. East Afr J Public Health. 2004; 1: 11–17.

[9] Jowett M, Miller N, Mnzava N. Malaria Expenditure Analysis: Tanzania Case Study. Report prepared for DFID-EA (Tanzania) and the Roll Back Malaria Initiative York: International Programme for Centre for Health Economics, University of York 2000.

[10] Saxena P, Reyburn H, Njau B, Chonya S, Mbakilwa H, Mills A. Patient costs for paediatric hospital admissions in Tanzania: a neglected burden? Health Policy Plan. 2010; 15: 328–333. 10.1093/heapol/czq003 20129938

[11] Hausmann Muela S, Mushi AK, Muela Ribera J. The paradox of the cost and affordability of traditional and government health serrvices in Tanzania. Health Policy Plan. 2000; 15: 296–302. 11012404 10.1093/heapol/15.3.296

[12] Mboera LEG, Senkoro KP, Mayala BK, Rumisha SF, Rwegoshora RT, Mlozi MRS, et al Spatio-temporal variation in malaria transmission intensity in five agro-ecosystems in Mvomero district, Tanzania. Geospat Health. 2010; 4: 167–178. 20503186 10.4081/gh.2010.198

[13] Tanzania Turiani-Hospital Foundation. Turiani Hospital, health for all in the Mvomero district, Masterplan May 2007.

[14] Filmer D, Pritchett LH. Estimating wealth effects without expenditure data–or tears: an application to education enrollments in states of India. Demography. 2001; 38: 115–132. 11227840 10.1353/dem.2001.0003

[15] Vyas S, Kumaranayake L. Constructing socio-economic status indices: how to use principal components analysis. Health Policy Plan. 2006; 21: 459–468. 17030551 10.1093/heapol/czl029

[16] Ministry of Health and AMMP team. Policy implications of adult morbidity and mortality: end of phase one report. Dar es Salaam: AMMP/Ministry of Health. 1997.

[17] Aikaeli J. Determinants of Rural Income in Tanzania: An Empirical Approach. Research Report 10/4. Dar es Salaam: Research on Poverty Alleviation. 2010.

[18] Alilio MS, Kitua A, Njunwa K, Medina M, Rønn AM, Mhina J, et al Malaria control at the district level in Africa: the case of the Muheza District in northeastern Tanzania. Am J Trop Med Hyg. 2004; 71: 205–213. 15331839

[19] McIntyre D, Thiede M, Dahlgren G, Whitehead M. What are the economic consequences for households of illness and of paying for health care in low- and middle-income country contexts? Soc Sci Med. 2006; 62: 858–865. 16099574 10.1016/j.socscimed.2005.07.001

[20] WHO, UNICEF. Integrated Management of Childhood Illness chart booklet Geneva: WHO Press 2008.

[21] Kalyango JN, Rutebemberwa E, Karamagi C, Mworozi E, Ssali S, Alfven T, et al High adherence to antimalarials and antibiotics under integrated community case management of illness in children less than five years in eastern Uganda. PLoS One. 2013; 8: e60481 10.1371/journal.pone.0060481 23555980 PMC3612059

