# Correction: Out-of-Pocket Costs and Other Determinants of Access to Healthcare for Children with Febrile Illnesses: A Case-Control Study in Rural Tanzania

**DOI:** 10.1371/journal.pone.0128166

**Published:** 2015-05-08

**Authors:** The PLOS ONE Staff

There are errors in the Funding section that were not corrected during the typesetting process. The correct funding information is as follows: This work was supported by the Sall Family Foundation via the Pan American Health and Education Foundation (renamed PAHO Foundation) and EUROHEALTH. The funders had no role in study design, data collection and analysis, decision to publish, or preparation of the manuscript. URL: http://www.pahofoundation.org.

The publisher apologizes for these errors.

## References

[1] Castellani J, Mihaylova B, Evers SMAA, Paulus ATG, Mrango ZE, Kimbute O, et al (2015) Out-of-Pocket Costs and Other Determinants of Access to Healthcare for Children with Febrile Illnesses: A Case-Control Study in Rural Tanzania. PLoS ONE 10(4): e0122386 doi:10.1371/journal.pone.0122386 25861012 10.1371/journal.pone.0122386 PMC4393118

