# Supplementary material for: Out-of-Pocket Costs and Other Determinants of Access to Healthcare for Children with Febrile Illnesses: A Case-Control Study in Rural Tanzania
Source: PLoS One. 2015 Apr 10;10(4):e0122386. doi: 10.1371/journal.pone.0122386 (PMC4393118; doi:10.1371/journal.pone.0122386)
Supplement: S1 Table — (DOC) [file pone.0122386.s001.doc]

**S1 Table. Mean private costs (US Dollar) for a whole episode of febrile illness for the cases by location, stratified by household wealth category.**

| **MEAN PRIVATE COSTS** | | | | |
| --- | --- | --- | --- | --- |
|  | | **(N)** | **TOTAL Cost (SD)** | **Cases Turiani vs Cases Kilosa: p value** |
| **Cases – Kilosa** | **Most poor** | 14 | 0.56 (0.72) |  |
|  | **Poor** | 10 | 1.67 (2.37) |  |
|  | **Least poor** | 8 | 2.43 (3.42) |  |
| **Cases – Turiani** | **Most poor** | 8 | 16.05 (4.95) | p<0.0001 |
|  | **Poor** | 7 | 10.43 (6.18) | p=0.0009 |
|  | **Least poor** | 4 | 11.08 (5.73) | p=0.0076 |
| **Total Cases** | **Most poor** | 22 | 6.20 (8.16) |  |
|  | **Poor** | 17 | 5.28 (6.10) |  |
|  | **Least poor** | 12 | 5.31 (5.88) |  |

Test for trend across wealth category: Kilosa p=0.042, Turiani: p=0.066, Overall p=0.719
