# Supplementary material for: Out-of-Pocket Costs and Other Determinants of Access to Healthcare for Children with Febrile Illnesses: A Case-Control Study in Rural Tanzania
Source: PLoS One. 2015 Apr 10;10(4):e0122386. doi: 10.1371/journal.pone.0122386 (PMC4393118; doi:10.1371/journal.pone.0122386)
Supplement: S2 Table — (DOC) [file pone.0122386.s002.doc]

**S2 Table. Determinants of private hospital costs for an episode of febrile illness based on a generalized linear model for 249 participants interviewed at the hospital.**

| **Variable** | **Covariates** | **Multiplicative effect¹** | **95% CI** | **p-value** |
| --- | --- | --- | --- | --- |
| Community | Kilosa | reference | - | - |
|  | Turiani | 3.124 | 2.485 – 3.927 | p<0.0001 |
| Severity of febrile episode | *Per Os* | reference | - | - |
|  | *Non Per Os* | 0.977 | 0.732 – 1.305 | 0.876 |
|  | Severe | 0.984 | 0.704 – 1.377 | 0.927 |
| Age of child (months) | - | 1.001 | 0.992 – 1.010 | 0.865 |
| Gender | Male | reference | - | - |
|  | Female | 0.863 | 0.693 – 1.075 | 0.189 |
| Wealth index* | - | 0.994 | 0.918 – 1.076 | 0.881 |
| Number of days spent at the hospital | - | 1.124 | 1.045 – 1.209 | 0.002 |

¹These are the exponentiated coefficients of the GLM model with log-link function between the linear predictor and mean private cost. The reference private cost of a 20-month-old male child from a family with an average Household wealth admitted in Kilosa hospital for 2.9 day and of; Per Os severity of illness throughout the episode is a median US$4.76.

* The higher the score, the better the wealth.
